# The Impact of Amyloid-Beta Positivity with 18F-Florbetaben PET on Neuropsychological Aspects in Parkinson’s Disease Dementia

**DOI:** 10.3390/metabo10100380

**Published:** 2020-09-23

**Authors:** Seunghee Na, Hyeonseok Jeong, Jong-Sik Park, Yong-An Chung, In-Uk Song

**Affiliations:** 1 Department of Neurology, Incheon St. Mary’s Hospital, The Catholic University of Korea, Seoul 21431, Korea; seunghee.na@gmail.com (S.N.); 77jjongsik@hanmail.net (J.-S.P.); 2 Department of Radiology, Incheon St. Mary’s Hospital, The Catholic University of Korea, Seoul 21431, Korea; hsjeong@catholic.ac.kr; 3 Department of Nuclear Medicine, Incheon St. Mary’s Hospital, The Catholic University of Korea, Seoul 21431, Korea

**Keywords:** Parkinson’s disease dementia, Alzheimer’s disease, positron emission tomography, executive function, neuropsychiatric symptoms

## Abstract

The neuropathology of Parkinson’s disease dementia (PDD) is heterogenous, and the impacts of each pathophysiology and their synergistic effects are not fully understood. The aim of this study was to evaluate the frequency and impacts of co-existence with Alzheimer’s disease in patients with PDD by using 18F-florbetaben PET imaging. A total of 23 patients with PDD participated in the study. All participants underwent 18F-florbetaben PET and completed a standardized neuropsychological battery and assessment of motor symptoms. The results of cognitive tests, neuropsychiatric symptoms, and motor symptoms were analyzed between the positive and negative 18F-florbetaben PET groups. Four patients (17.4%) showed significant amyloid burden. Patients with amyloid-beta showed poorer performance in executive function and more severe neuropsychiatric symptoms than those without amyloid-beta. Motor symptoms assessed by UPDRS part III and the modified H&Y Scale were not different between the two groups. The amyloid PET scan of a patient with PDD can effectively reflect a co-existing Alzheimer’s disease pathology. Amyloid PET scans might be able to help physicians of PDD patients showing rapid progression or severe cognitive/behavioral features.

## 1. Introduction

Parkinson’s disease (PD) is a common neurodegenerative disorder related to accumulation of alpha-synuclein and loss of neurons in the substantia nigra. Cognitive impairment in PD is common, and approximately 80% of patients with PD cumulatively develop dementia along with disease progression [1,2]. However, the neuropathology of Parkinson’s disease dementia (PDD) is heterogeneous, including levels of alpha-synuclein, amyloid-beta, and tau protein. The impacts of each pathophysiology and their synergistic effects have not been fully understood until now [3,4,5]. It is known that development of dementia in PD is associated with widespread Lewy bodies from the brainstem to the limbic and isocortex [1,6], and up to half of PDD patients also showed co-existence of amyloid-beta and tau pathology, fulfilling the diagnosis of Alzheimer’s disease [4,5].

Amyloid-beta positron emission tomography (PET) has enabled visualization of the amyloid pathology in vivo and is widely used to identify Alzheimer’s disease in clinical practice [7]. The amyloid-beta PET shows a positive scan in patients with significant amyloid burden regardless of cognitive function, even in cognitively healthy subjects. Thus, the impact of positive results in amyloid-beta PET should be interpreted carefully. In a longitudinal follow-up, co-existing amyloid burden detected via florbetapir PET had a relation to faster progression to significant cognitive impairment in patients with PD [8]. We hypothesized that Alzheimer’s disease might affect the behavioral and psychological symptoms and frontal/executive functions in patients with PDD. Thus, we evaluated the frequency of Alzheimer’s disease using 18F-florbetaben PET imaging and the impact of co-existence with Alzheimer’s disease on neuropsychological aspects in patients with PDD.

## 2. Results

**Figure 1:**
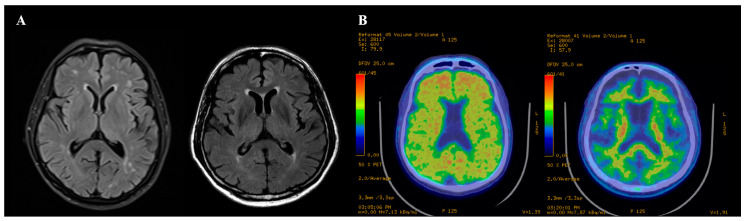
The representative images. (**A**) The brain MRI. The brain MRI images of the patients with amyloid burden (Left) and without amyloid burden (Right) were unremarkable. (**B**) The 18F-fluorbetaben PET. The left image showed significant amyloid plaque deposition in the cerebral cortex. The right image revealed a negative finding.

Twenty-three patients were included and had a mean age of 73.7 ± 5.7 years. Mean scores of the modified H&Y scales and the UPDRS part III were 2.2 ± 1.0 and 15.8 ± 8.8, respectively. Four of 23 patients (17.4%) with PDD revealed positive amyloid PET scan (Aβ+), and the others were negative (Aβ−) (Figure 1). The demographic and clinical characteristics of the patients with PDD are summarized in Table 1. Demographics of age, sex, and educational level between the Aβ+ group and Aβ− group were not significantly different. The scores of the modified H&Y scale and the UPDRS part III and administered LEDD at the scan time did not differ between the two groups. There was no significant difference in MMSE or CDR-SB. The scores of NPI were significantly higher in the patients with positive amyloid PET scan (7.2 ± 13.0 vs. 32.0 ± 37.0, *p* = 0.037). In neuropsychological testing, scores of the COWAT, a method evaluating executive function, were significantly lower in patients with Aβ+ PET. The remaining results of the neuropsychological testing were not different between the two groups (Table 2).

## 3. Discussion

This study showed that 17.4% (4/23) of patients with PDD showed a positive amyloid PET scan. It is also revealed that PDD patients with significant amyloid burden demonstrated as Aβ+ PET were associated with severe behavioral symptoms and impairment in executive function.

Our result is in line with previous works. The frequency of positivity on amyloid PET in PDD was reported as 17–33% [9,10,11], similar to that in PD without dementia [12,13]. In patients with PDD, individuals with positive 18F-florbetapir PET imaging also harbored autopsy-confirmed Aβ-positive plaques in multiple brain regions, which were correlated with regional florbetapir binding [14]. Post-mortem studies showed parenchymal amyloid-beta deposition in one quarter of patients with PD [15] and 32–55% of those with PDD [15,16,17].

A strong association between overall amyloid-beta deposition and accumulation of cortical alpha-synuclein, a key pathology forming Lewy bodies, has been reported in patients with PD [18]. In Lewy body disease with dementia, brains with significant amyloid-beta deposition showed extensive alpha-synuclein inclusions and higher concentrations of insoluble alpha-synuclein [19]. Brains of patients with AD showed higher levels of alpha-synuclein pathology in regions with more frequent neuritic plaques than in those with moderate or sparse plaques [20]. A previous study with an animal model demonstrated that double transgenic mice with amyloid-beta and alpha-synuclein showed much more severe and earlier cognitive impairment and more abundant alpha-synuclein inclusions than single, alpha-synuclein transgenic mice [21]. Some of the alpha-synuclein inclusions in double transgenic mice were fibrillar, whereas all alpha-synuclein inclusions in single alpha-synuclein transgenic mice were amorphous. That is, the amyloid-beta burden in Parkinson’s disease may promote the accumulation, aggregation, and misfolding of alpha-synuclein, resulting in pathology.

In general, patients with PDD are characterized by behavioral features and cognitive impairments in executive functions, visuospatial functions, and memory [22]. Even without dementia, patients with PD often present with executive dysfunction, seeming to have resulted from nigrostriatal and mesocortical dopamine denervation [23]. It is also suggested that cholinergic and noradrenergic deficit occurred in conjunction with dopaminergic depletion and associated with impaired episodic memory and executive function [23]. Our results show that patients with PDD and Aβ+ revealed higher scores on the NPI and lower scores on the COWAT. A previous longitudinal follow-up study demonstrated a faster decline in executive function in patients with PDD who showed florbetapir (+) PET [24]. Episodic memory function, the main feature of Alzheimer’s dementia, was not different between Aβ+ and Aβ-PET groups in patients with PDD [24]. Similarly, in 115 PD patients with normal cognition, mild cognitive impairment (MCI), or dementia, there was no difference in various measures of global cognitive scores and memory scores according to the status of amyloid deposition assessed by Florbetaben PET imaging [25]. Another study showed indistinguishable neuropsychological aspects between PDD patients with or without amyloid-beta pathology [17]. On the other hand, in patients with PD-MCI, PiB uptake was significantly related to poor performance in global cognitive measures and the Wechsler Adult Intelligence Scale (WAIS) [13].

We found that PDD patients with Aβ+ scored significantly higher in NPI than subjects with Aβ-. Neuropsychiatric symptoms such as depression, apathy, and hallucination are common in PDD [26]. In an autopsy study, patients with PD and an Alzheimer’s type pathology demonstrated significantly higher frequency of neuropsychiatric symptoms, including depression and psychosis, than those without an AD pathology. Patients with PDD and an AD pathology showed more frequent hallucinations, but the difference was not significant [5]. Neuroimaging studies have revealed that behavioral and psychological symptoms including apathy and hallucination in PDD are related to altered metabolism in the frontal lobe [26], the key structure for executive function, in which patients with PD showed early prominent dysfunction. Moreover, our results reveal that the scores of UPDRS-part III and modified H&Y scale did not differ significantly between Aβ+ and Aβ− groups. These findings are similar to previous works; the prevalence and severity of motor symptoms of patients with PDD and amyloid-beta pathology were not significantly different compared to those in patients without amyloid-beta [5,10,17].

In postmortem studies of the impact of Alzheimer’s pathology, patients with PD with pathology-confirmed AD had been diagnosed with PD at a relatively older age than those who did not [4,17,27]. In addition, PD patients with pathology-confirmed AD also had a higher risk of developing dementia than those without it [27]. Moreover, patients with PD with an AD pathology also showed shorter disease duration and poor survival compared to those without AD [4,27]. Combinations of various neuropathologies including alpha-synuclein and amyloid-beta and tau showed better discrimination power of dementia than single neuropathology [27]. Patients with PD and an amyloid pathology were associated with a shorter duration between symptom onset and diagnosis of dementia [17,28]. Thus, the AD pathology in PD may contribute to and act synergistically with an alpha-synuclein pathology on the timing of diagnosis in patients with dementia and deteriorations in cognitive function.

This study has several limitations. The number of participants was small, although the results are in line with previous reports. Although participants were recruited prospectively, this is a cross-sectional study. Thus, our results show correlations, not causality, of neuropsychiatric aspects and a specific cognitive task with the positivity of amyloid PET scan.

In conclusion, amyloid PET scan of a patient with PDD can effectively reflect co-existing Alzheimer’s disease pathology. Patients with PDD and co-existing amyloid-beta showed poorer performance on executive function and more severe neuropsychiatric symptoms than those without amyloid-beta. Amyloid PET scans might be able to help physicians of PDD patients showing rapid progression or severe cognitive/behavioral features. Understanding the underlying pathology could motivate more effective treatment strategy and prediction of disease prognosis. Further studies with more patients and longitudinal follow-up are needed.

## 4. Materials and Methods

### 4.1. Subjects

We consecutively recruited patients with PDD who underwent an amyloid PET scan at Incheon St. Mary’s Hospital from November 2018 to January 2019. Parkinson’s disease was diagnosed based on the clinical diagnostic criteria of the UK Brain Bank for PD [29]. All of the patients with PDD fulfilled the Movement Disorder Society consensus criteria for probable PDD [22]. The diagnosis of PDD was made after at least one year from the time of diagnosis with PD. We obtained informed consent from participants before enrollment. The study was approved by the Institutional Review Board of Incheon St. Mary’s Hospital (OC18OESI0025).

### 4.2. Clinical and Neuropsychological Assessments

Demographic data, levodopa equivalent daily dose (LEDD), the Unified Parkinson’s Disease Rating Scale (UPDRS) motor scores, and results of amyloid PET scan were collected. The motor portion of the UPDRS (item number 18–31, range = 0–108, higher scores indicating more severe symptoms) for the severity of PD symptoms was assessed by a neurologist (I.U.S.). Each LEDD was calculated according to the standard method as previously reported [30].

The Clinical Dementia Rating (CDR) scale, the CDR-sum of boxes (CDR-SB), and neuropsychiatric assessments including Geriatric Depression Scale-Short form (SGDS) and Neuropsychiatric Inventory (NPI) were evaluated. All patients underwent the standardized Seoul Neuropsychological Screening Battery (SNSB) [31], which included Mini-Mental State Examination (MMSE), digit span forward and backward, Controlled Oral Word Association Test (COWAT) with phonemic and category (animals) fluency, Stroop test, the Rey Complex Figure Test (RCFT), the Korean version of the Boston Naming Test (K-BNT), and delayed recall and recognition tasks from the Seoul Verbal Learning Test (SVLT) and RCFT. The neuropsychological testing was evaluated in the patient’s on state, within 1 month of amyloid PET scan. The Clinical Dementia Rating-Sum of Box (CDR-SB) was also calculated.

### 4.3. PET Imaging

All patients underwent PET/CT with an F-18 florbetaben imaging session using a hybrid PET/CT Discovery system (General Electric Medical Systems, Milwaukee, WI, USA). Patients received an intravenous injection of 296 MBq ± 10% of F-18 florbetaben. The dose was administered as a single bolus injection followed by 20 cc of saline flush. Patients rested in a waiting room after F-18 florbetaben injection. Image acquisition started approximately 90 min after injection, with a scan duration of 15 min. Once ready for the study, the patient was positioned in the scanner. A CT scan (helical, kV = 120 kV, mA = 20, reconstructed slice thickness = 2.5 mm) was acquired for attenuation correction of PET data. Three-dimensional (3D) PET acquisition (List Mode) started 90 min after 18F-florbetaben injection. Image reconstruction was performed using a 3D-OSEM algorithm with the following parameters: Image matrix = 128, Field of View = 250 mm, Subsets = 24, Iterations = 3, Post Filter = 3 mm Full Width Half Maximum, Attenuation Correction = CT-based. Acquired images were interpreted by a reader who was blinded to clinical information of patients according to a previously described method [32] and classified the result as either positive or negative.

### 4.4. Statistical Analysis

The Mann–Whitney test and chi-square test were used to compare differences in demographics, clinical characteristics, and results of neuropsychological battery for continuous and categorical variables, respectively. The level of statistical significance was set at *p* < 0.05. All statistical analyses were performed using the SPSS software package, version 18.0.

## Figures and Tables

**Table 1:** Demographic and clinical characteristics.

	Aβ−	Aβ+	
	*n* = 19	*n* = 4	*p* Value
Age	73.4 ± 6.0	74.8 ± 4.6	0.8574
Female	5 (26.3%)	2 (50.0%)	0.5573
H&Y stage	2.3 ± 1.1	1.6 ± 0.5	0.3164
UPDRS part III	16.6 ± 9.2	11.8 ± 5.7	0.2422
LEDD (mg)	786 ± 338	600 ± 491	0.5128
Educational level (years)	8.3 ± 5.3	8.5 ± 2.6	0.9904
MMSE	23.1 ± 4.5	21.0 ± 6.4	0.4069
CDR	0.6 ± 0.2	0.9 ± 0.8	0.6717
CDR-SB	2.2 ± 2.1	4.1 ± 4.7	0.4305
GDS-SF	5.2 ± 3.6	8.0 ± 7.0	0.5407
NPI	7.2 ± 13.0	32.0 ± 37.0	0.0366

Values are shown as mean ± standard deviation or number (%). H&Y: Modified Hoehn and Yahr Scale; UPDRS: Unified Parkinson’s Disease Rating Scale; LEDD: Levodopa Equivalent Daily Dose; MMSE: Mini-Mental State Examination; CDR: Clinical Dementia Rating scale; CDR-SB: Clinical Dementia Rating scale-sum of boxes; GDS-SF: Geriatric Depression Scale-Short Form; NPI: neuropsychiatric inventory.

**Table 2:** Results of neuropsychological test.

	Aβ−	Aβ+	
	*n* = 19	*n* = 4	*p* Value
Digit span forward	5.9 ± 1.6	6.5 ± 2.1	0.528
Digit span backward	2.7 ± 1.5	2.8 ± 0.5	0.713
COWAT-animal	10.2 ± 3.5	6.5 ± 3.8	0.095
COWAT-phonemic	13.0 ± 8.0	3.0 ± 2.4	0.013
Stroop test	48.5 ± 27.5	41.7 ± 22.2	0.748
Rey complex figure copy	25.4 ± 10.0	21.4 ± 14.4	0.442
Boston naming test	35.5 ± 12.8	27.5 ± 9.1	0.171
SVLT delayed recall	2.7 ± 2.4	0.5 ± 0.6	0.114
RCFT delayed recall	5.6 ± 6.5	1.6 ± 3.2	0.327

Values are shown as mean ± standard deviation. COWAT: Controlled Oral Word Association Test; SVLT: Seoul Verbal Learning Test; RCFT: Rey Complex Figure Test.
